# Comment on “Choroidal Thickness in Patients with Mild Cognitive Impairment and Alzheimer's Type Dementia”

**DOI:** 10.1155/2016/8737082

**Published:** 2016-06-28

**Authors:** Abdullah Ilhan, Umit Yolcu, Fahrettin Akay

**Affiliations:** ^1^Ophthalmology Service, Ankara Mevki Military Hospital, 06110 Ankara, Turkey; ^2^Ophthalmology Service, Izmir Military Hospital, 35150 Izmir, Turkey

The authors congratulate Bulut et al. [1] for their study entitled “Choroidal Thickness in Patients with Mild Cognitive Impairment and Alzheimer's Type Dementia.” The authors investigated choroidal and macular thickness in Alzheimer's type dementia (ATP), mild cognitive impairment (MCI), and control healthy subjects. The findings are impressive and show that choroidal thickness in the subfoveal, nasal, and temporal retinal regions are all significantly thinner in ATP and MCI patients with respect to control eyes. However, the authors did not find any significant change in terms of macular thickness. The findings are really important, because longitudinal choroidal thickness data may be used in suspected patients.

We realized that the authors have included both eyes of the participants in the statistical analyses. We want to emphasize that including both eyes of participants in the statistical analyses violates the independence of the variables when using ANOVA and/or independent samples *t*-test. Although that is true, we think that including only one eye of participants will not possibly affect the results, because the differences are really big between the groups. We want to ask the authors whether they have retinal nerve fiber layer thickness and/or ganglion cell complex data of the patients and make statistics between the groups. It is plausible that any central nervous system pathology may initially affect retinal nerve fibers compared to other ocular structures including choroid.

In addition, we see that the authors investigated the correlations between mini-mental state examination (MMSE) and choroidal thickness by including all the participants. We kindly ask the authors to perform these statistics in each group. The result of those statistics can tell us whether MMSE may be used in the follow-up of patients in terms of progression analysis.
